# The prognostic significance and immune correlation of SLC10A3 in low-grade gliomas revealed by bioinformatic analysis and multiple immunohistochemistry

**DOI:** 10.18632/aging.204712

**Published:** 2023-05-10

**Authors:** Yang He, Junlin Song, Yong Qin, Dejia Mao, Dacheng Ding, Shanwu Wu, Huawei Wu

**Affiliations:** 1 Department of Neurosurgery, Sinopharm Dongfeng General Hospital, Hubei University of Medicine, Shiyan 442000, Hubei, China; 2 Department of Neurosurgery, Affiliated Hospital of Zunyi Medical University, Zunyi Medical University, Zunyi 563000, Guizhou, China; 3 Department of Neurosurgery, Wuhan Forth Hospital, Wuhan 430000, Hubei, China

**Keywords:** low-grade gliomas, multiplex immunohistochemistry, immunotherapy, correlation analysis, immune microenvironment

## Abstract

Purpose: Despite the fact that genetic risk factors contribute to low-grade gliomas (LGGs), the role of critical genes as prognostic and theraputic biomarkers is quite limited. This study is designed to comprehensively investigate the prognostic role and predictive ability of solute carrier family 10 member 3 (*SLC10A3*) for immunotherapy in LGGs.

Methods: We analyzed the prognostic value of *SLC10A3* from multiple datasets of LGG patients, and explored its immune correlation via multiple algorithms. Finally, we independently confirmed the clinical significance and its immune correlation using the multiplex staining assay of LGG samples on the tissue microarray.

Results: *SLC10A3* mRNA was up-regulated in LGGs compared with normal brain tissues, and correlated with tumor grade, histological type, IDH wide type and non-codel 1p19q. Up-regulation of SLC10A3 transcription was remarkably associated with shortened overall survival time compared with down-regulation in TCGA, CGGA and Rembrandt datasets, and *SLC10A3* exhibited good predictive ability for survival outcomes among LGGs. Correlation analyses showed that *SLC10A3* mRNA expression correlates well with the six immune check points and immune cells. When the expression and immune correlation of *SLC10A3* at the translational level were verified via multiplex immunohistochemistry, expression of *SLC10A3* protein was higher in LGG compared with normal tissues, and expression of *SLC10A3* protein was correlated well with macrophage, CD4 + T cell and B cell.

Conclusions: Up-regulation of *SLC10A3* mRNA is statistically associated with adverse survival outcomes and immune infiltration among LGGs. *SLC10A3* might be a reliable survival predictor and a promising immunotherapy target for LGG patients.

## INTRODUCTION

Low-grade gliomas (LGGs) are slow-growing intracranial tumors with the tendency for the transformation of high-grade glioma. LGGs seriously reduce patients’ quality of life both due to the lesion in brain and the side effects of treatment [1]. The utility of molecular feature for LGGs is recommended in the 2021 WHO guideline, which highly emphasizes the prognostic role of IDH1 mutation in LGGs [2]. The standard treatment for LGGs is surgical resection along with postoperative chemoradiotherapy, and the survival outcomes of LGG individuals are generally better than high-grade glioma population [3]. Besides, immunotherapy, which regulates the tumor immune microenvironment, has been successfully utilized in patients with melanoma, lung cancer, and even gliomas [4]. However, not all the individuals with LGGs benefit from immunotherapy, so accurate selection of immunotherapies requires a comprehensive understanding of the underlying tumor immune phenotype.

The solute carrier (SLC) family 10 (SLC10) encodes protein for transporters of various agents, bile acids and steroidal hormones, and SLC01A3 belongs to the SLC10 family [5]. *SLC10A3*, located in an unmethylated CpG island, is a 4 kb gene. *SLC10A3*, correlated to the human X chromosome, is a housekeeping protein [6]. *SLC10A3* consists of 477 amino acids, with a calculated mass of approximately 50 kDa, but the functions of *SLC10A3* protein are largely unknown. Chen et al. [7] used the two next-generation sequencing (NGS) technologies to reveal that *SLC10A3* expression was closely correlated to the resistance of chemotherapy in ovarian cancer. A recent research performed by Tian et al. [8] uncovered the clinical relevance and immune correlation of *SLC10A3* in liver cancer. Our bioinformatic analysis unveiled that expression of *SLC10A3* in LGGs is highly correlated with the infiltration abundance of immune cells and the expression of immune checkpoint. Hence, systematically illustrating role of *SLC10A3* in the tumor immune microenvironment is conducive to identify a novel therapeutic target for LGGs.

It is significant to evaluate the abnormal changes in *SLC10A3* expression and the relationship between *SLC10A3* expression and survival outcomes and immune cells by conducting an integrative analysis before the wild application of *SLC10A3* in the clinical practice. In this work, we initially determined the differential expression of *SLC10A3* in LGG tissues and normal brain specimens. Then we explored the role of *SLC10A3* mRNA in the survival assessment among LGG patients from TCGA, CGGA and Rembrandt datasets. Importantly, due to the controversial survival significance of *SLC10A3* RNA in LGG, we undertook meta-analysis to determine the overall prognostic significance of *SLC10A3* in LGG. Subsequently, we correlated the *SLC10A3* expression and immune association in the LGG dataset. Finally, we employed the multiple immunohistochemistry (mIHC), a sophisticated assay of multiplex immunofluorescence staining, which allows simultaneous detection of a panel of target proteins, to determine the immune microenvironment and ascertain the correlation between *SLC10A3* expression and immune checkpoints (PD-1 and PD-L1) at the protein level.

## RESULTS

### SLC10A3 expression is up-regulated in LGGs

**Figure 1:**
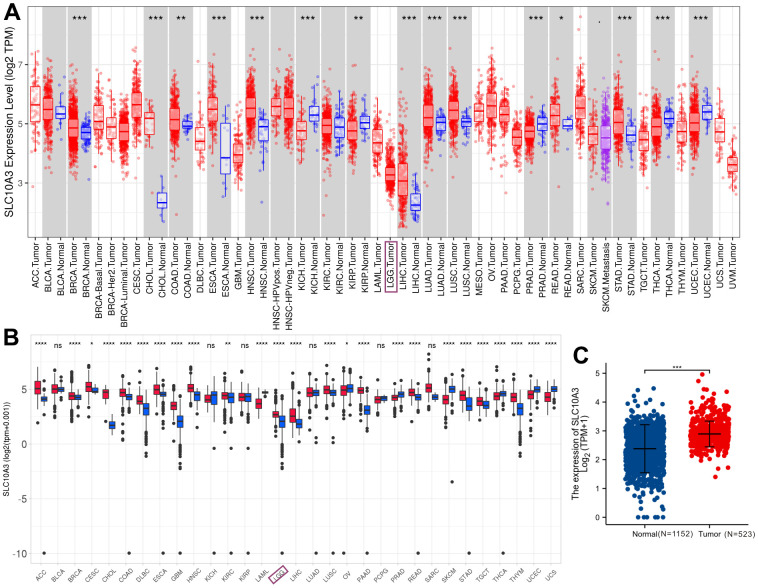
**Expression levels of *SLC10A3* transcription across pan-cancer and LGG.** (**A**) Expression profiles of SLC10A3 mRNA across pan-cancer tissues and normal tissues based on TCGA data. (**B**) Expression profiles of *SLC10A3* transcription in pan-cancer tissues and normal tissues based on TCGA and GTEx data. (**C**) Expression levels of SLC10A3 are up-regulated in LGG tissues compared with normal tissues.

We initially mined TIMER database to investigate the expression of *SLC10A3* transcription in pan-cancer and LGGs. As displayed in Figure 1A, we found that *SLC10A3* is up-regulated in most types of malignant tumors, such as breast cancer, cholangiocarcinoma, esophageal carcinoma, head and neck squamous cell carcinoma, liver cancer, lung cancer, colorectal cancer, gastric cancer. While *SLC10A3* is down-regulated in renal cancer, uterine corpus endometrial carcinoma and thyroid carcinoma. Due to the lack of normal brain tissues from TCGA-LGG dataset, we could not know whether there existed the difference of *SLC10A3* expression between LGGs and normal brain tissues. Thereby, we further explored the Shiny database, which is mainly based on the TCGA data and GTEx database. In line with the results from TIMER, we still observed that *SLC10A3* expression was up-regulated in most malignant tumors, including LGGs (Figure 1B). Next, we downloaded the original gene expression data of TCGA-LGG (N=523) and GTEx (N=1152), and the comparison was also significant (Figure 1C).

### Relationship between *SLC10A3* expression and clinical features of LGGs

**Figure 2:**
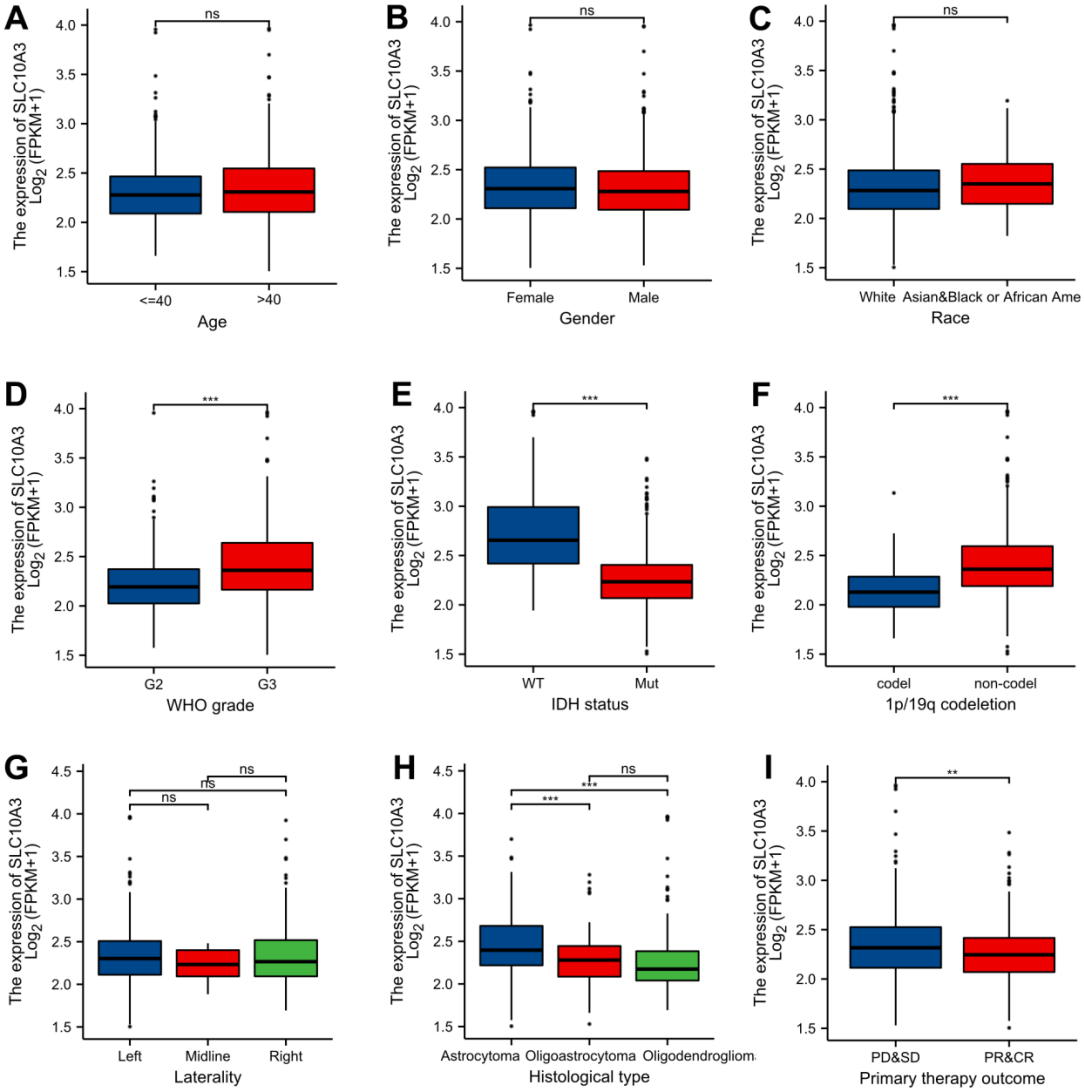
**Distribution of *SLC10A3* transcription among different clinical groups.** (**A**) age. (**B**) gender. (**C**) race. (**D**) WHO grade. (**E**) IDH mutation status. (**F**) 1p19q co-deletion. (**G**) laterality. (**H**) histological type. (**I**) primary therapy outcome.

We compared the *SLC10A3* mRNA expression in different clinical groups. As exhibited in Figure 2A–2I, we found that *SLC10A3* expression was significantly higher in LGGs with G3 (P<0.001), IDH wide type (P<0.001), non-codel 1p19q (P<0.0001), astrocytoma (P<0.001) and progression (P<0.01). Hence, due to the close association between SLC10A3 and worse clinical factors, we inferred that *SLC10A3* serves as an oncogene in LGGs.

### Down-regulation of *SLC10A3* is a reliable prognostic indicator for LGGs

**Figure 3:**
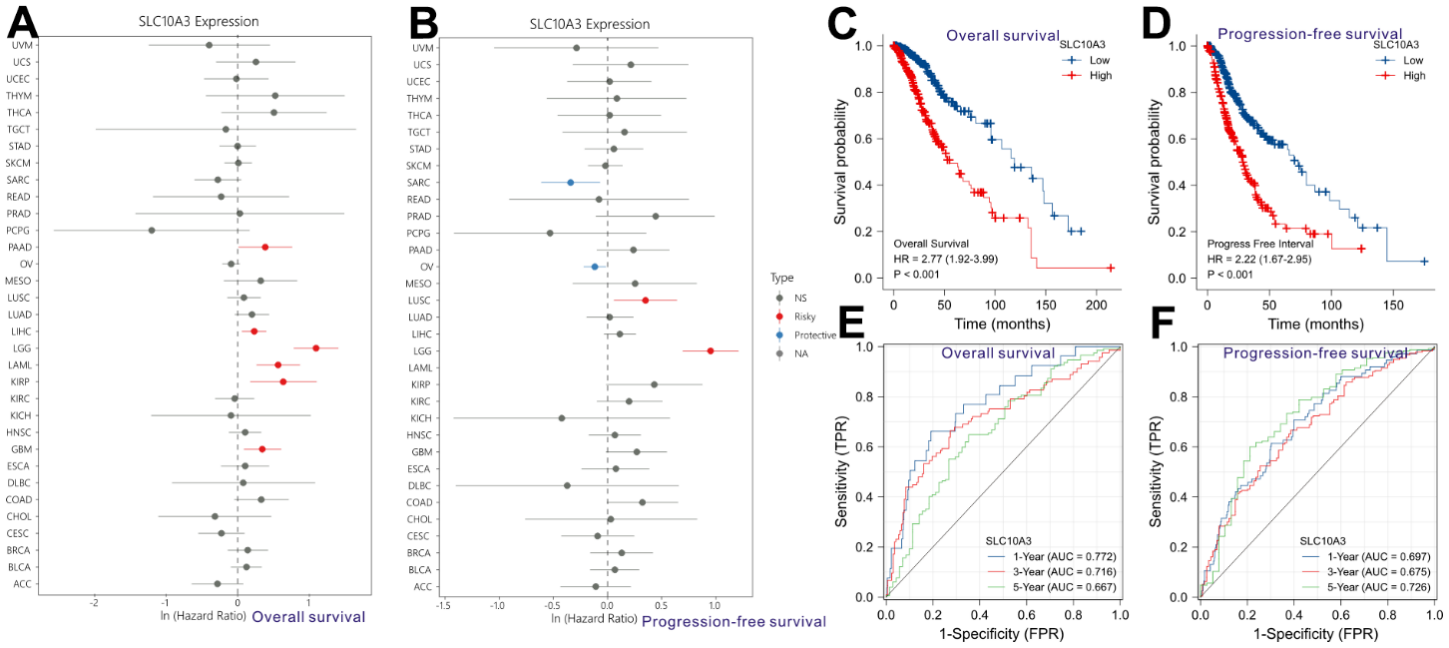
**Prognostic role of *SLC10A3* in pan-cancer and LGG.** Single-factor Cox regression analysis reveals that up-regulation of *SLC10A3* is a risk factor for poor overall survival (**A**) and progression-free survival (**B**) among LGG individuals. Survival curves display that down-regulated of *SLC10A3* is correlated with favorable overall survival (**C**) and progression-free survival (**D**) among LGG individuals. *SLC10A3* showed nice performance for the prediction of 1-year, 3-year, 5-year overall survival rate (**E**) and progression-free survival (**F**) rate for LGG individuals.

Pan-cancer analysis revealed that up-regulation of *SLC10A3* is not only a risk factor for worse overall survival among LGGs (Figure 3A), but also a risk factor for inferior progression-free survival among LGGs (Figure 3B). We classified these LGG individuals into low *SLC10A3* group and high *SLC10A3* group based on the median expression value of *SLC10A3*. According to the survival curves, up-regulation of *SLC10A3* transcription is remarkably associated with shortened overall survival time compared with down-regulation (HR=2.77, 95%CI:1.92-3.99, P<0.0001, Figure 3C). Similarly, up-regulation of *SLC10A3* transcription is remarkably associated with shortened progression-free survival time compared with down-regulation (HR=2.22, 95%CI:1.67-2.95, P<0.0001, Figure 3D). Next, we drew the td-ROC curves to measure the predictive ability of *SLC10A3* expression for the 1-year, 3-year and 5-year survival rate of LGG individuals. The expression of *SLC10A3* reached good predictive performance for overall survival rate (1-year AUC: 0.772, 3-year AUC:0.716, 5-year AUC:0.667, Figure 3E), and for progression-free survival (1-year AUC: 0.697, 3-year AUC:0.675, 5-year AUC:0.726, Figure 3F).

**Figure 4:**
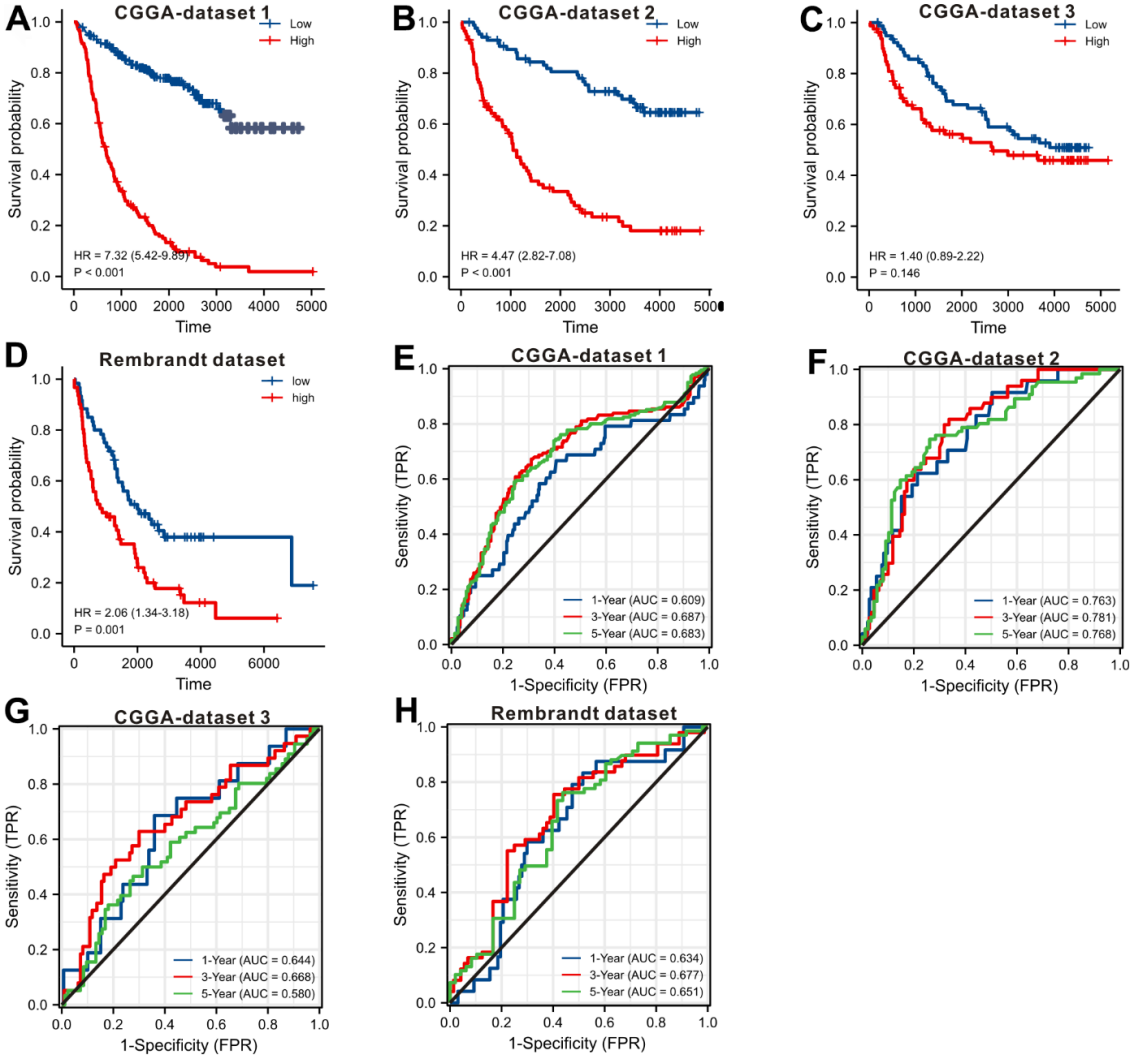
**Validating the prognostic value of *SLC10A3* with other LGG datasets.** Survival curves display that down-regulated of *SLC10A3* is correlated with favorable overall survival in CGGA dataset 1 (**A**), CGGA dataset 2 (**B**), CGGA dataset 3 (**C**), and Rembrandt dataset (**D**), *SLC10A3* showed nice performance for the prediction of 1-year, 3-year, 5-year overall survival rate in CGGA dataset 1 (**E**), CGGA dataset 2 (**F**), CGGA dataset 3 (**G**), and Rembrandt dataset (**H**).

In order to externally confirm the prognostic significance, we also explored the CGGA database and Rembrandt dataset. We downloaded the original gene expression data and only retained the LGGs’ data. The survival curves (Figure 4A–4D) displayed that up-regulation of *SLC10A3* in LGGs is also a risk prognostic factor in the four LGG datasets, but the difference was not significant in CGGA dataset 3. So we carried out the meta-analysis via STATA software to confirm whether up-regulation of *SLC10A3* transcription is a risk prognostic indicator for LGGs. As listed in Supplementary Figure 1, up-regulation of *SLC10A3* transcription is remarkably correlated with less favorable overall survival among LGG population (HR=3.07, 95%CI:1.68-5.63, P<0.0001). Subsequently, we also explored the predictive ability of *SLC10A3* expression for survival rate, and we found that *SLC10A3* expression still exhibited nice predictive performance for 1-year, 3-year and 5-year survival rate based on four LGG datasets (Figure 4E–4H).

### Immune correlation with *SLC10A3* expression in LGGs

The extent of immune cell infiltration in tumor areas is an important risk factor affecting the survival outcomes of LGGs [9]. Single-factor Cox regression provided by TIMER database showed that *SLC10A3* expression and infiltration of immune cells were the prognostic factors of LGGs (Supplementary Figure 2A–2F), and multiple Cox regression analysis displayed that higher expression of *SLC10A3* and macrophage infiltration were the independent risk indicators for less favorable overall survival among patients with LGGs (Supplementary Table 1). Furthermore, we focused our attention on the relationship between immune cell infiltration and *SLC10A3* expression. Correlation plots displayed that expression of *SLC10A3* was closely linked with the infiltration of immune cells, such as B cells (r=0.456, P=6.28*10^-26^, Supplementary Figure 2G), CD8+ T cells (r=0.25, P=3.08*10^-8^, Supplementary Figure 2H), CD4 +T cells (r=0.547, P=1.7*10^-38^, Supplementary Figure 2I), tumor associated macrophage (TAM) (r=0.562, P=9.36*10^-41^, Supplementary Figure 2J), neutrophil (r=0.606, P=6.19*10^-49^, Supplementary Figure 2K) and dendritic cell (r=0.593, P=1.67*10^-46^, Supplementary Figure 2L).

**Figure 5:**
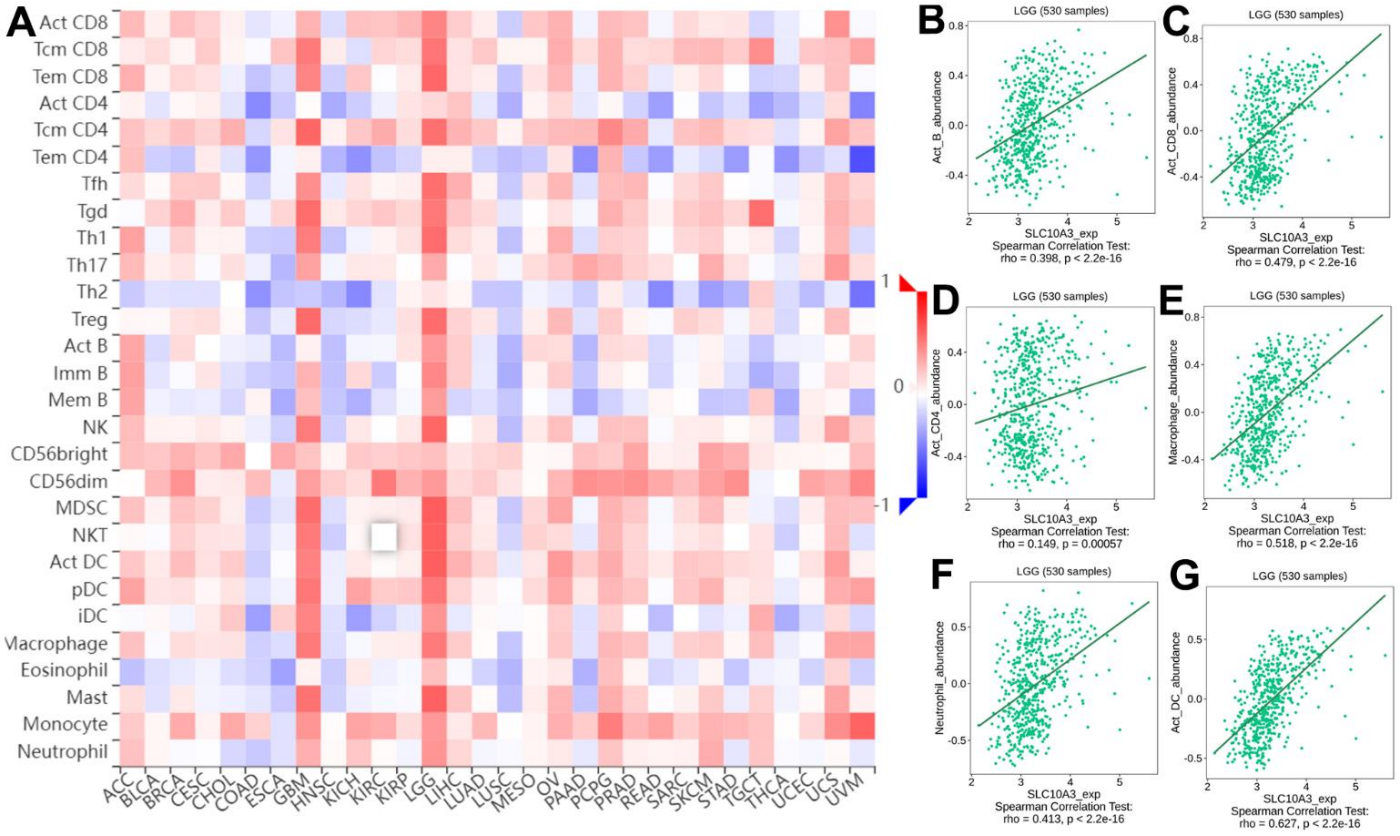
**Correlation between infiltrations of immune cells and expression of *SLC10A3* in pan-cancer and LGG.** (**A**) Heat map displays expression of *SLC10A3* is correlated well with most immune cells in LGG and GBM. Expression of *SLC10A3* is highly associated with the infiltrations of B cells (**B**) CD8+T cells (**C**) CD4+T cells (**D**) macrophages (**E**) neutrophils (**F**) and dendritic cells (**G**) in LGG.

**Figure 6:**
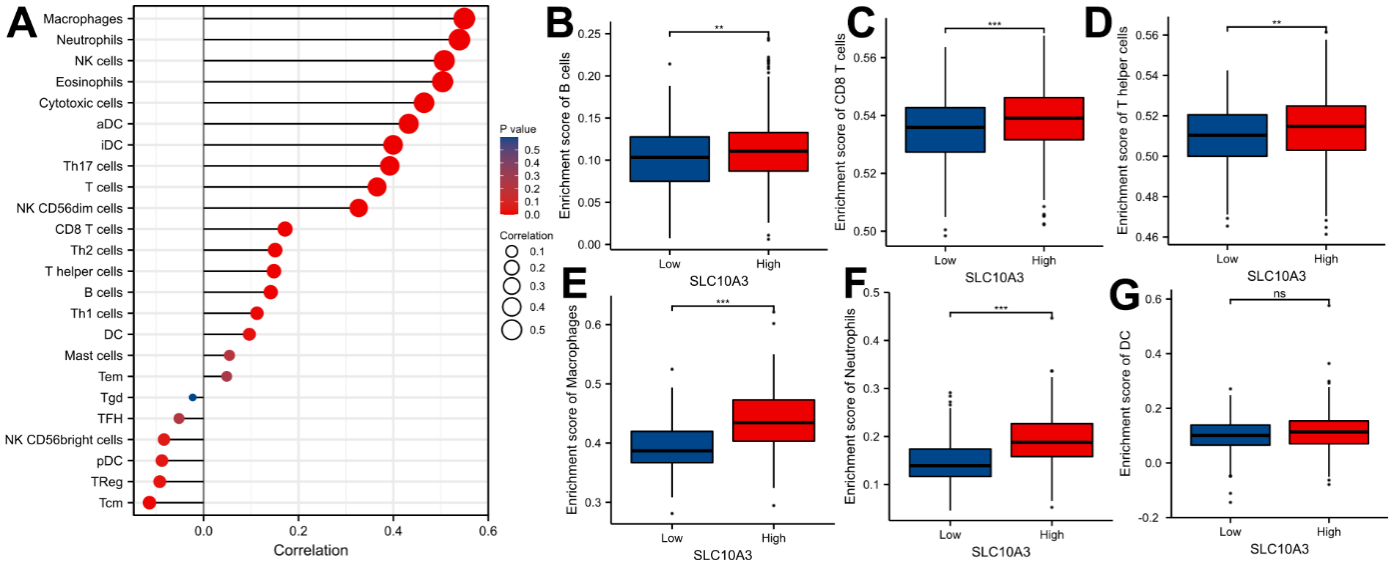
**Distribution of *SLC10A3* in various of immune cells in LGG.** (**A**) Correlation analyses of *SLC10A3* and 24 immune cells measured by ssGSEA in LGG. Expression of *SLC10A3* is significantly different subgroups divided by B cells (**B**) CD8+T cells (**C**) T helper cells (**D**) macrophages (**E**) neutrophils (**F**) and dendritic cells (**G**) in LGG.

We searched the TISIDB database to further confirm the relationship between *SLC10A3* expression and immune cells in LGG. Figure 5A exhibited the relationship between *SLC10A3* expression and immune cells in pan-cancer, and the most close relationship was seen in LGG and GBM. The detailed correlation plots also verified the strong relationship between *SLC10A3* expression and immune cells in LGGs (Figure 5B–5G). We assessed the distribution of *SLC10A3* mRNA in different immune types of LGG, and statistical analysis proved that distribution of *SLC10A3* mRNA was different among the four immune types (P=1.01*10^-19^, Supplementary Figure 3A), with C3 (inflammatory) exhibiting the highest *SLC10A3* mRNA. As for the six molecular types of LGG, results of one-way ANOVA showed that distribution of SLC10A3 was significantly different among the six molecular types of LGG (P=6.84*10^-26^, Supplementary Figure 3B), with 1p19q codel displaying the lowest *SLC10A3* expression. Finally, we employed the ssGSEA to explore the correlation of *SLC10A3* with most types of immune cells in LGGs. As shown in Figure 6A, expression of *SLC10A3* is strongly associated with infiltration of macrophages, followed by neutrophils, which was almost consistent with the correlation results from TIMER database. When we divided the LGG individuals into high *SLC10A3* and low *SLC10A3* groups, we noticed that the distributions of immune cells were almost different between high *SLC10A3* and low *SLC10A3* groups except for dendritic cells (Figure 6B–6G).

### Correlation analysis between *SLC10A3* and immune check points in LGGs

We selected TIMER algorithm to quantify the association between *SLC10A3* expression and six immune check points. Strong relationships between *SLC10A3* expression and PD-1 (r=0.568, P=2.29e^-45^, Supplementary Figure 4A), PD-L1 (r=0.478, P=8.98e^-31^, Supplementary Figure 4B), PD-L2 (r=0.549, P=6.74e^-42^, Supplementary Figure 4C), HAVCR2 (r=0.585, P=9.63e^-49^, Supplementary Figure 4D), IDO1(r=0.433, P=5.16e-^25^, Supplementary Figure 4E) and LAG3 (r=0.348, P=4.1e^-16^, Supplementary Figure 4F). Next, we browsed the TISIDB database for further verification. Fortunately, we also noticed the positive relationships between *SLC10A3* expression and the six immune check points in LGG (Supplementary Figure 3C–3H). In a word, *SLC10A3* expression correlates well with the six immune check points, indicating that *SLC10A3* might occupy an important role in the regulation of immunotherapy.

In the whole ICIs cohort, we did not observe the statistical difference of *SLC10A3* expression between responders and non-responders (Supplementary Figure 5A). When we classified the population into primary and recurrent tumor individuals, and the expression of *SLC10A3* was not significantly different between the two subgroups among primary tumor population (Supplementary Figure 5B), but the expression of *SLC10A3* was remarkably different between the two subgroups among recurrent tumor population (Supplementary Figure 5C). We drew the ROC plots to quantify the predictive ability of *SLC10A3* to immunotherapy. The predictive capacity of *SLC10A3* was 0.505 in the whole cohort (Supplementary Figure 5D), 0.507 in the primary tumor cohort (Supplementary Figure 5E) and 0.674 in the recurrent tumor cohort (Supplementary Figure 5F). We noticed that the predictive capacity of *SLC10A3* was the highest among recurrent tumor population, most likely to the fact that they exhibited the higher expression of *SLC10A3*, and up-regulation of *SLC10A3* correlating well with the immune check points.

### Biological pathways of *SLC10A3* in LGGs

We only selected the co-expressed genes with |logFC|>2 and *P*<0.0001 for the subsequent enrichment analysis. After running with clusterProfiler package, the biological processes of *SLC10A3* in LGG were identified. KEGG pathways revealed that *SLC10A3* was most likely involved in cytokine-cytokine receptor interaction, viral protein interaction with cytokine and cytokine receptor and transcriptional misregulation in cancer (Supplementary Figure 6A). The detailed genes involved in biological pathways and KEGG pathways were listed in Supplementary Figure 6B.

### Validation of the association at translational level via mIHC

**Figure 7:**
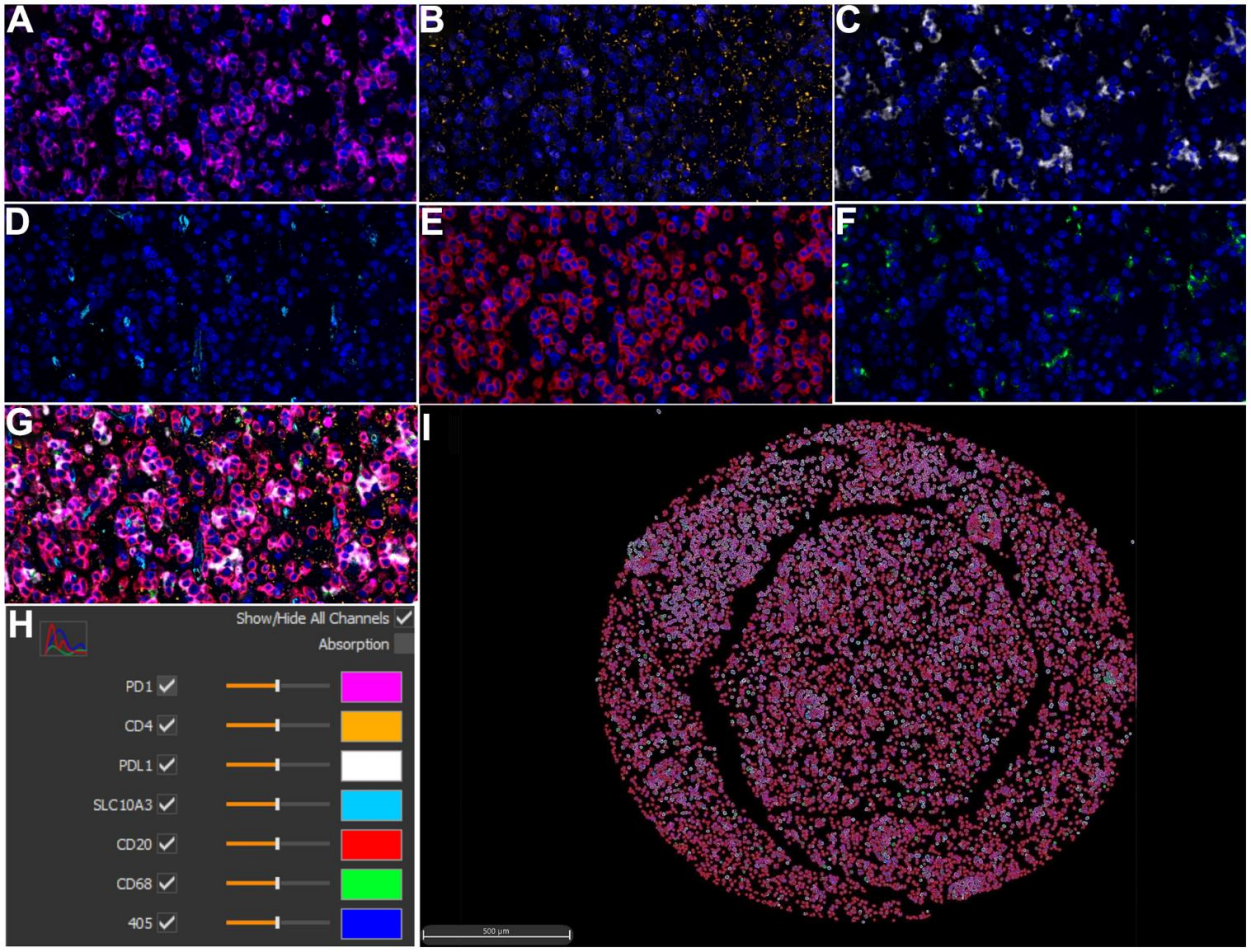
**Multiplex immunohistochemistry profiling of *SLC10A3* and immune markers in LGG.** (**A**) PD1 (pink), (**B**) CD4 (yellow), (**C**) PD-L1 (white), (**D**) *SLC10A3* (blue). (**E**) CD20 (red), (**F**) CD68 (green). (**G**) The merged image of seven markers. (**H**) Each marker stands for one special color. (**I**) Cell phenotype image constructed by the seven markers in the multiplex staining.

To further confirm the above immune correlations at translational level for clinical pathology, an advanced method of mIHC was utilized to detect the protein expression of *SLC10A3* and the selected immune markers on LGG tissues (Figure 7A–7I) and normal tissues (Supplementary Figure 7A–7I). Then, we compared the detailed parameters of *SLC10A3* between LGG and normal tissues. As shown in Supplementary Figure 8A–8I, we noticed that number of *SLC10A3* positive cells is more in LGG tissues than normal tissues in total area and tumor area, and *SLC10A3* average cell intensity was stronger in LGG tissues than normal tissues in stroma area. Subsequently, we analyzed the correlation between *SLC10A3* expression and immune markers at the translational level via Spearman correlation. As listed in Table 1, we observed that *SLC10A3* expression was correlated well with macrophage, CD4 T cell and CD20 B cell in LGG, which is almost consistent with the correlation result from genetic level.

**Table 1:** Correlations analyses of SLC10A3 and immune markers in LGG detected by mIHC.

**Area**	**Indexes**	**PD1**	**CD4**	**PD-L1**	**CD20**	**CD68**
Total area	SLC10A3 Positive Cells	0.295	0.245	0.268	0.031	0.474**
	SLC10A3 Avg Positive Cytoplasm Intensity	-0.048	0.436**	-0.044	-0.083	0.186
	SLC10A3 Avg Cell Intensity	0.293	0.451**	0.264	0.627**	0.579**
Tumor area	SLC10A3 Positive Cells	0.255	0.249	0.2	-0.067	0.485**
	SLC10A3 Avg Positive Cytoplasm Intensity	-0.048	0.413*	-0.066	-0.173	0.163
	SLC10A3 Avg Cell Intensity	0.296	0.481**	0.295	0.651**	0.602**
Stromal area	SLC10A3 Positive Cells	0.259	0.333*	-0.147	0.175	0.363*
	SLC10A3 Avg Positive Cytoplasm Intensity	0.105	0.394*	0.024	0.108	0.264
	SLC10A3 Avg Cell Intensity	-0.225	0.17	-0.172	0.023	0.515**

## DISCUSSION

This is the first report to systematically unveil the expression profile, prognostic role, immune association and biological pathways of *SLC10A3* in LGGs. Our analysis identified that *SLC10A3* mRNA was highly expressed in LGG tissues than the normal brain tissues. Survival analysis from multiple datasets highlighted that over-expression of *SLC10A3* mRNA is significantly linked to worse overall survival among LGGs except for Rembrandt dataset. Hence, we undertook the meta-analysis to ensure whether *SLC10A3* is a prognostic factor in LGG patients, and the overall survival significance represented by HRs from 1490 LGGs proved our hypothesis. The close relationship between *SLC10A3* expression and various immune cells is observed in LGG tissues, which is not only calculated by TIMER but also validated by ssGSEA algorithms. Finally, we utilized the multiple IHC assay to test the expression of several immune cell markers and immune check points (PD-1 and PD-L1) in LGG tissues and corresponding normal tissues. The experimental results proved the close association between *SLC10A3* protein and macrophages infiltration in LGG tissues. In summary, our analysis demonstrated that *SLC10A3* is an oncogene and may play an important role in the immune response in LGG.

Tumor-infiltrating immune cells belong to the complex tumor microenvironment and may occupy an important role in the tumorigenesis [10]. Tumorigenesis and tumor metastasis are characterized by immune evasion, which is a major obstacle to effective therapy against LGGs [11]. A thorough understanding of the immune microenvironment of LGG is crucial to enhance the efficacy of current Immune checkpoint blockers [12]. The mIHC, a novel biotechnology, provides multiplex staining and standardized quantitative analysis for highly cost-effective tissue studies when compared with the conventional IHC assay. The fast development of multiple immunofluorescences leads to the successful application of mIHC, which makes it possible to detect a panel of fluorescent proteins on one tissue microarray (TMA) slide within the same time. Huang et al. used the mIHC assay to reveal that spatial heterogeneity of macrophage in gastric cancer [13]. Zhang et al. [14] applied this novel technology identified CD133 and PD-L1 as diagnostic biomarkers for colorectal cancer (CRC) with early-stage, while they identified the HER2 as an advanced biomarker for CRC. To our knowledge, this is the first research to report the application of mIHC in LGGs. The mIHC results showed that *SLC10A3* protein is correlated well with the abundance of TAMs in the stromal areas of LGGs, which is consistent with the results from transcription level based on data from TCGA-LICH dataset. Moreover, our results revealed *SLC10A3* acts as the malignant characters in LGGs partly via the enrichment with TAMs.

MIHC is widely considered to play an increasingly important role in the field of cancer immunotherapy [15]. Lu et al. [16] found that mIHC is correlated with better performance in the prediction of response to PD-1 or PD-L1 treatment in malignant tumors when compared to tumor mutational burden, IHC alone. Joe et al. [17] clarified that mIHC can simultaneously quantify PD-L1 labeling with multiple antibodies, and allow accurate analysis of immune cells and immune check markers, which serves a promising assay in the era of immunotherapy. Our bioinformatic analysis discovered that expression of *SLC10A3* transcription is highly linked to the expression of PD-1 based on different algorithms. The further mIHC assay also confirmed the relationship in stromal tissues of LGGs. Moreover, enrichment analysis also demonstrated that *SLC10A3* co-expressed genes are involved in antigen binding and immunoglobulin complex. We inferred that *SLC10A3* plays the important role in the regulation of immunotherapy in LGGs, which might be a potential biomarker for the response to immunotherapy in LGGs.

LGGs are generally divided into two subgroups based on IDH mutation status due to the different survival outcomes, while LGG individuals with EGFR amplification usually experienced poor prognosis. The well-established prognostic markers of LGGs include IDH mutation, MGMT methylation and 1p/19q codeletion [18]. With the clinical application of immunotherapy in LGGs, it’s quite necessary to develop novel survival indicators that could predict the immunotherapy response. In the present analysis, we found that low levels of *SLC10A3* transcription is significantly associated with better survival outcomes based on TGCA-LGG dataset. When validated with other LGG datasets, we found the survival significance is not unified. Meta-analysis is increasingly important in the field of cancer research [19, 20]. Meta-analysis is an easy-to-use method for combining the results from different researches, and this method is utilized to resolve conflicts among different studies [21]. Recently, meta-analysis has also been applied in resolving conflicts among different datasets [22]. Five public datasets related to LGGs were adopted in our analysis, and we used the Kaplan-Meier curves to assess the prognostic value of *SLC10A3* in LGGs. Except for CGGA dataset 3, low levels of *SLC10A3* transcription were significantly linked to favorable overall survival among LGGs. Hence, meta-analysis was performed to solve the conflicting issue, and the overall results depicting that *SLC10A3* is a reliable prognostic indicator for LGGs.

Although this is the first report related to the expression profiles, survival significance, immune correlation and biological functions of *SLC10A3* in LGGs, this analysis still existed three limitations. First, we could not directly calculate the performance of *SLC10A3* for the prediction of immunotherapy response among LGGs, due to the lack of LGGs cohort of immunotherapy. Then, our study is mainly based on the bioinformatic exploration and mIHC validation, but no cellular experiments are carried out to investigate the exact biological function. As for the mIHC validation, the included patients were only 36 cases. Except for the small sample size, we did not obtain the survival data. Thereby, our next research will focus on the exact biological mechanism of *SLC10A3* in glioma.

## CONCLUSIONS

*SLC10A3* mRNA and protein are up-regulated in LGGs compared with normal brain tissues, and the up-regulation of *SLC10A3* transcription is significantly correlated with adverse survival outcomes of LGGs individuals. *SLC10A3* plays a significant role in immune cell infiltration and regulation of immunotherapy response. Therefore, *SLC10A3* might be a reliable survival index and a promising immunotherapy target for LGG patients.

## MATERIALS AND METHODS

### Comprehensive bioinformatic analysis

TIMER database (https://cistrome.shinyapps.io/timer/, version 2.0) was mined to investigate the *SLC10A3* expression in pan-cancer, and to assess its immune correlation with a list of immune cells and immune check points. RNA expression data were utilized to assess the infiltration status of six immune cells (B cells, macrophages, CD8+ T cells, neutrophils, CD4+ T cells and dendritic cells) in LGG tissues. Spearman correlation analysis was selected to quantitatively measure the potential correlation between immune infiltration and SLC10A3 expression. Xena shiny website (https://shiny.hiplot.com.cn/ucsc-xena-shiny/, version 1.0 beta) was searched to explored the expression and prognostic value of *SLC10A3* expression in pan-cancer. We also downloaded the original gene expression data along with clinical information from UCSC Xena (http://xena.ucsc.edu/) and CGGA database (http://www.cgga.org). The clinical information of LGG individuals includes sex, race, age at diagnosis, IDH mutation, WHO grade, 1p19q codeletion, tumor site, histological type and treatment response. As the CGGA database contains three different glioma datasets, we renamed them as CGGA dataset 1(mRNAseq_693), CGGA dataset 2 (mRNAseq_325) and CGGA dataset 3 (mRNA-array_301), and we excluded the patients with high-grade gliomas. TISIDB database (http://cis.hku.hk/TISIDB/index.php) was explored to elucidate the *SLC10A3* expression and immune cell interplay in LGG. Furthermore, we adopted R software (version 3.6.3) to complete the ssGSEA via the GSVA package and enrichment analysis via the clusterProfiler package. ROC Plotter database (https://www.rocplot.org/) is able to link response to immunotherapy and gene expression using RNA-sequencing data of various malignant tumors. We selected the module of ROC Plotter for immunotherapy, and then entered SLC10A3. We downloaded the original results of immunotherapy and redrew the comparative plots and ROC curves.

### Sample collection

We collected sample tissues of LGG to verify the proteomic association between *SLC10A3* expression and immune cells (CD20 B cell, CD4 T cell, macrophage) and immune check markers (PD-1, PD-L1). We only included the primary LGG patients who experienced surgical resection. A total of 36 cases of LGG along with paracancerous samples during 2021-1 to 2022-1 were finally included in our study. We constructed the TMA based on the collected samples for the subsequent mIHC assay. Our study was proceeded under the approval of the Clinical Ethics Committee of Sinopharm Dongfeng general hospital (LW-2022-038), and all the procedures were conducted in line with the Declaration of Helsinki. All the patients gave their consent to this study prior to study commencement.

### Process of mIHC

In our experiment, TSA (Tyramide Signal Amplification) technology is utilized in in immunohistochemistry. By combining AlphaTSA 7-color fluorescent staining kit with multispectral imaging analysis technology, we successfully achieved fluorescence labeling and weak signal imaging. Specifically., the LGG TMA slide was deparaffinized by dimethylbenzene, followed by ethanol with different concentration. After rinsing, the LGG TMA slide was pre-treated with citric acid solution (pH 6.0) by microwaving method for antigen retrieval. Subsequently, the LGG TMA slide was blocked in 10% BSA blocking solution, and then stained with the primary antibodies against CD20, CD4, CD68, *SLC10A3*, PD-1 and PD-L1 (Supplementary Table 2), respectively for 1 h at room temperature. Each second antibody binds to a different fluorophore using AlphaTSA Multiplex IHC kit (No.AXT37100031, Beijing, China). Cell nucleus were finally stained with DAPI after all the six antigens being labeled. After mounting, the LGG TMA slide was imaged for quantitative analysis via ZEN software (version 3.3).

### Statistical analysis

All the related statistical tasks were performed using SPSS (version 18.0) and STATA (version 11.0). The relationships between *SLC10A3* expression and pathological features evaluated by chi-square test for categorical indexes, otherwise by the Wilcoxon rank sum test for continuous data. Kaplan-Meier curves were applied to compare the survival difference between high *SLC10A3* group and low *SLC10A3* group, and the survival difference was evaluated by log-rank test. The predictive efficacy of *SLC10A3* mRNA for 1-year, 3-year and 5-year overall survival rates was evaluated by time-dependent (td) receiver operating characteristic (ROC) curves. Correlation between *SLC10A3* with immune cells and immune check points were rated by Spearman correlation analysis. Meta-analysis was employed via STATA software to ensure the overall prognostic significance of *SLC10A3* in LGG individuals. As for meta-analysis of the overall prognostic effect, pooled HRs for survival along with its 95% CI were calculated by a fixed-effects model if there is no substantial heterogeneity across various LGG datasets; otherwise, a random-effects model was performed if there exists significant heterogeneity across various LGG datasets. Heterogeneity across various LGG datasets was assessed with the I^2^ statistic. *P* values less than 0.05 were deemed statistically significant.

### Data sharing statement

The datasets and original data used and/or analyzed during the current study are available from the corresponding authors on reasonable request.

## Supplementary Material

Supplementary Figures

Supplementary Tables
