# Supplementary material for: The prognostic significance and immune correlation of SLC10A3 in low-grade gliomas revealed by bioinformatic analysis and multiple immunohistochemistry
Source: Aging (Albany NY). 2023 May 10;15(9):3771–90. doi: 10.18632/aging.204712 (PMC10449292; doi:10.18632/aging.204712)
Supplement: Supplementary Figures [file aging-15-204712-s001.pdf]

SUPPLEMENTARY FIGURES

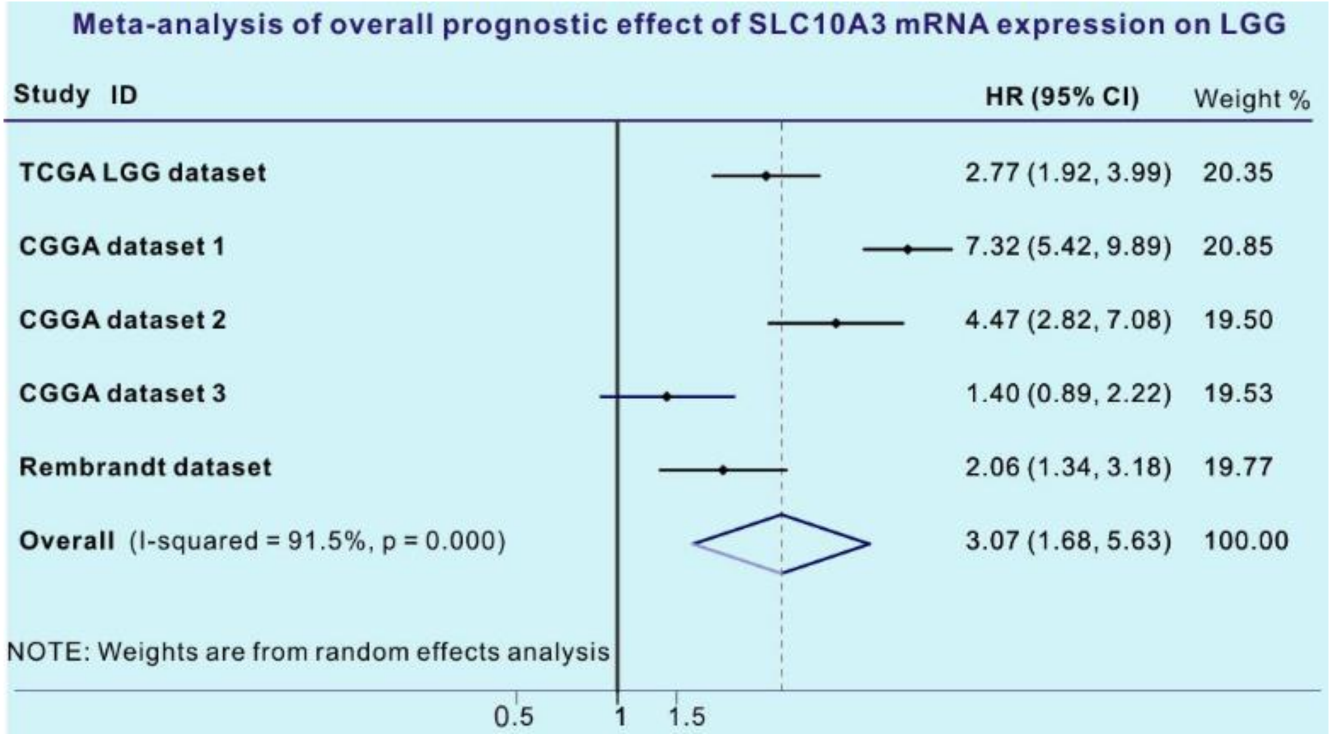

Supplementary Figure 1. Meta-analysis of the overall prognostic effect of *SLC10A3* among five LGG datasets.

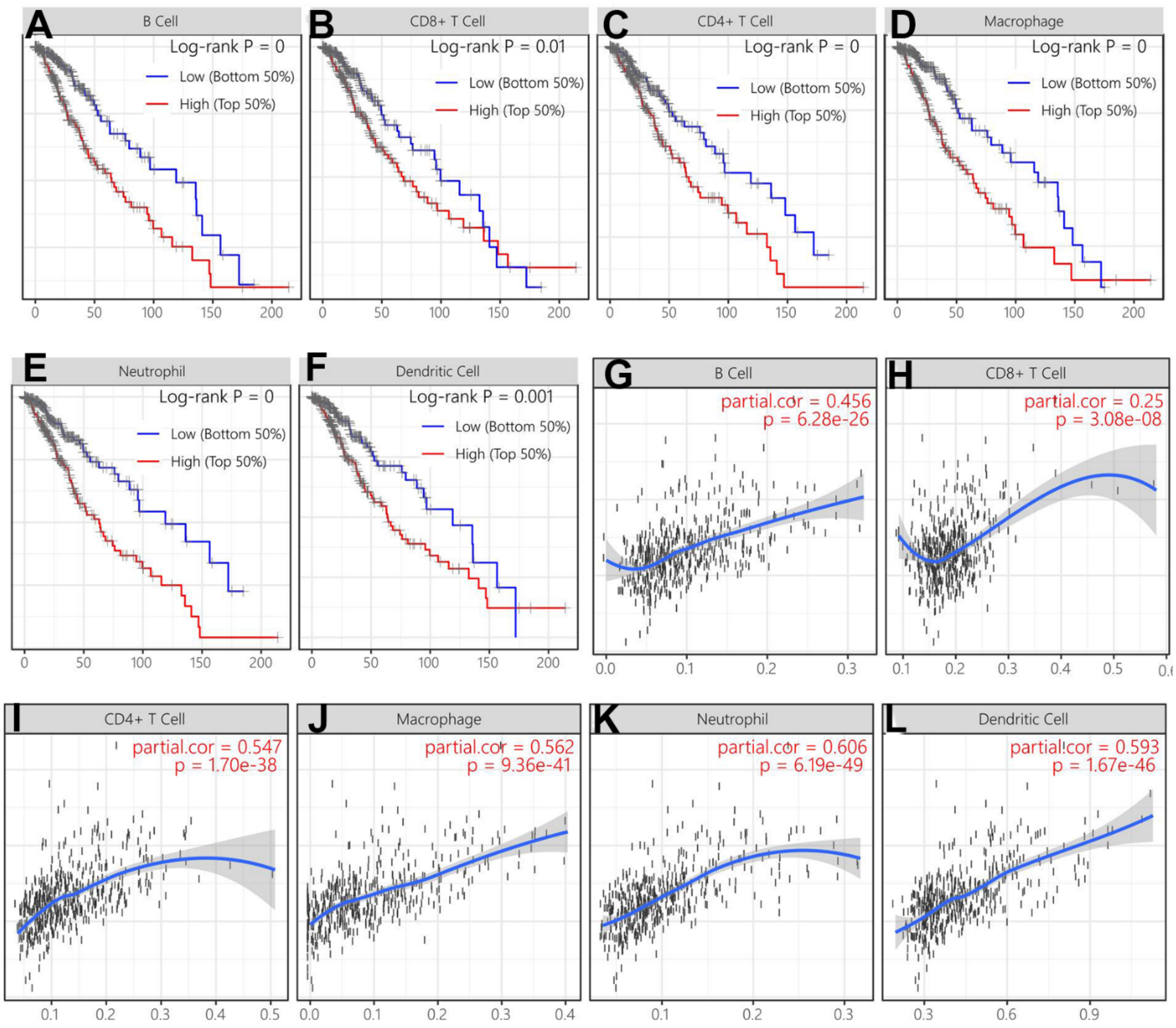

Supplementary Figure 2. Survival analysis of immune cells and correlation with *SLC10A3* expression in LGG. Low expression of immune cells. ((**A**) B cell, (**B**) CD8+T cell, (**C**) CD4+ T cell, (**D**) Macrophage, (**E**) Neutrophil, (**F**) Dentritic cell) is correlated with better overall survival in LGG. *SLC10A3* expression is positively associated with the abundance of immune cells ((**G**) B cell, (**H**) CD8+T cell, (**I**) CD4+ T cell, (**J**) Macrophage, (**K**) Neutrophil, (**L**) Dentritic cell).

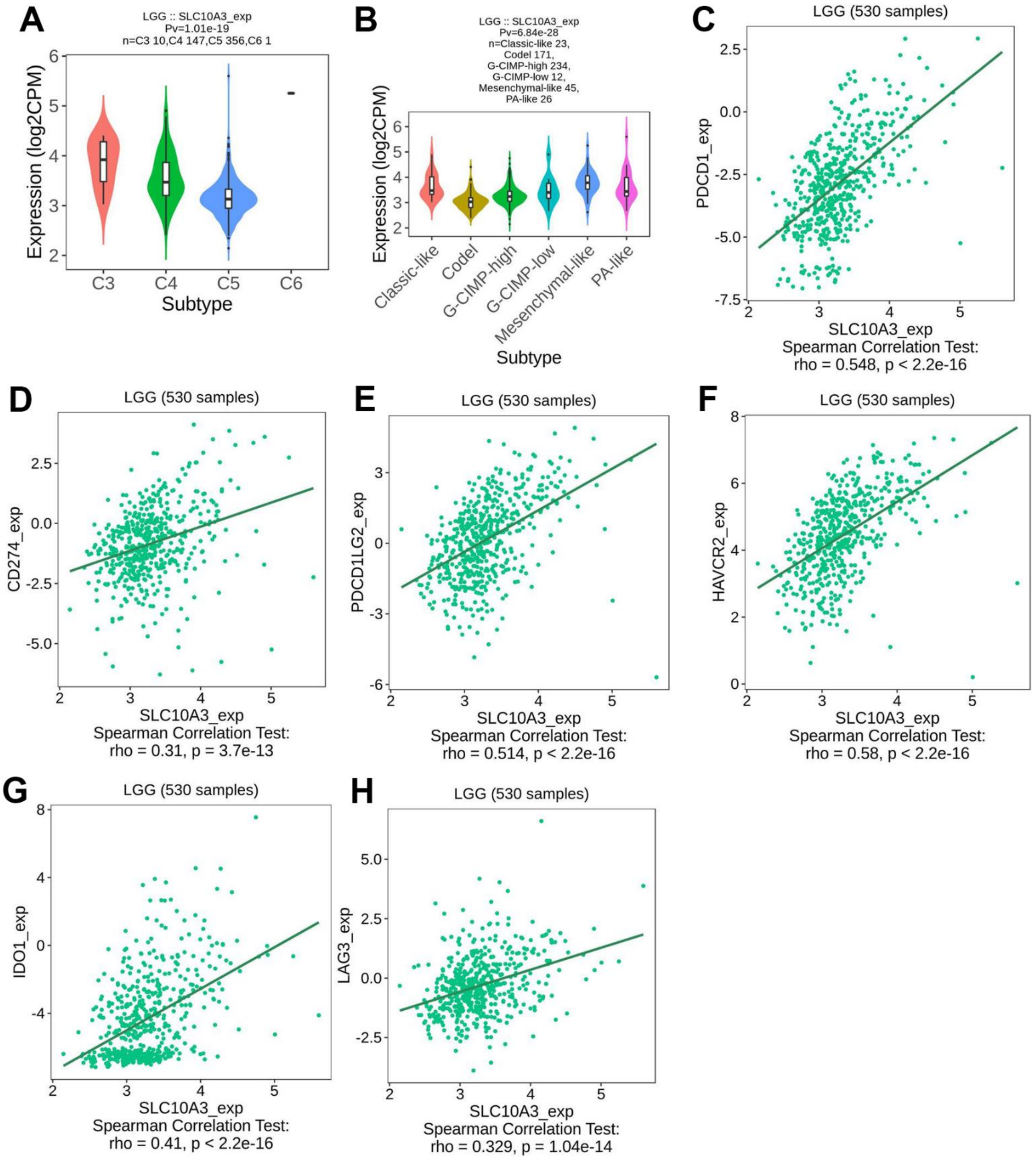

Supplementary Figure 3. Correlation of *SLC10A3* expression with immune sub-types and immune check points in LGG, which was obtained from TISIDB database. (**A**) Expression of *SLC10A3* is remarkably different among the four immune sub-types. (**B**) Expression of *SLC10A3* is remarkably different among the six molecular sub-types. Expression of *SLC10A3* is positively linked with immune check points ((**C**) PDCD1. (**D**) CD274. (**E**) PDCD1LG2. (**F**) HAVCR2. (**G**) IDO1. (**H**) LAG3).

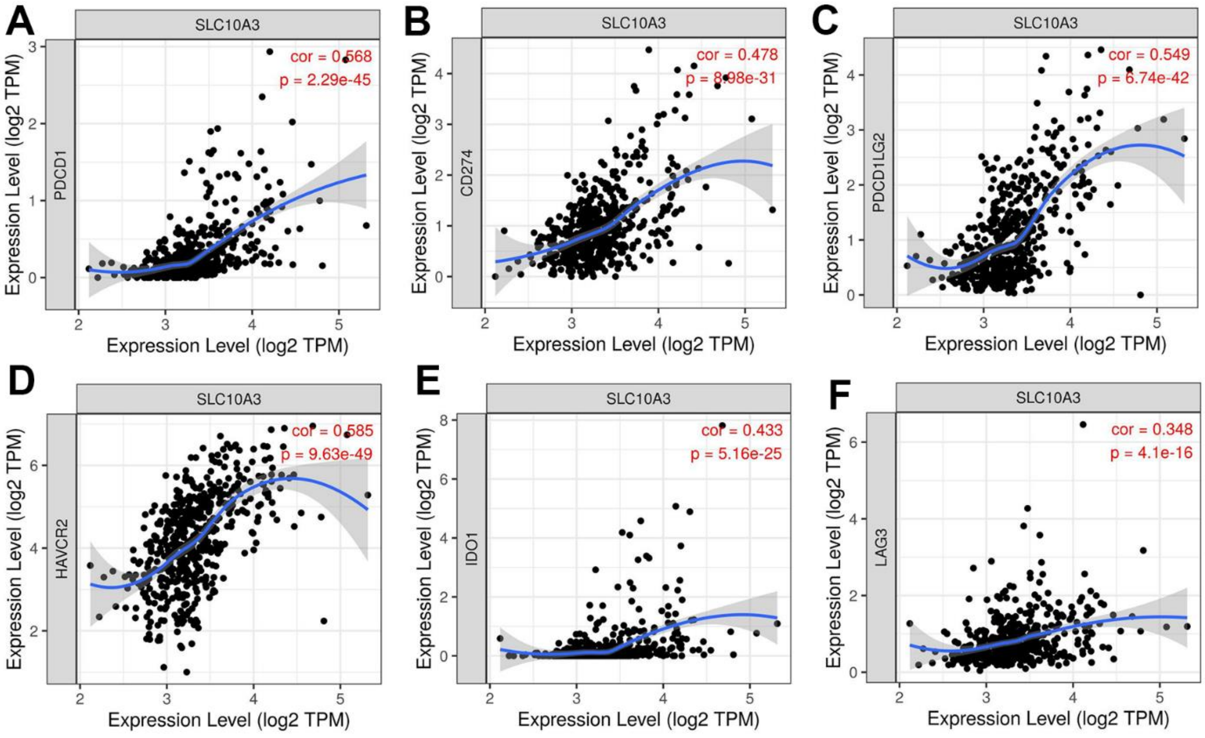

Supplementary Figure 4. Correlation analysis of immune check points and *SLC10A3* expression in LGG, which was obtained from TIMER database. (**A**) PDCD1. (**B**) CD274. (**C**) PDCD1LG2. (**D**) HAVCR2. (**E**) IDO1. (**F**) LAG3.

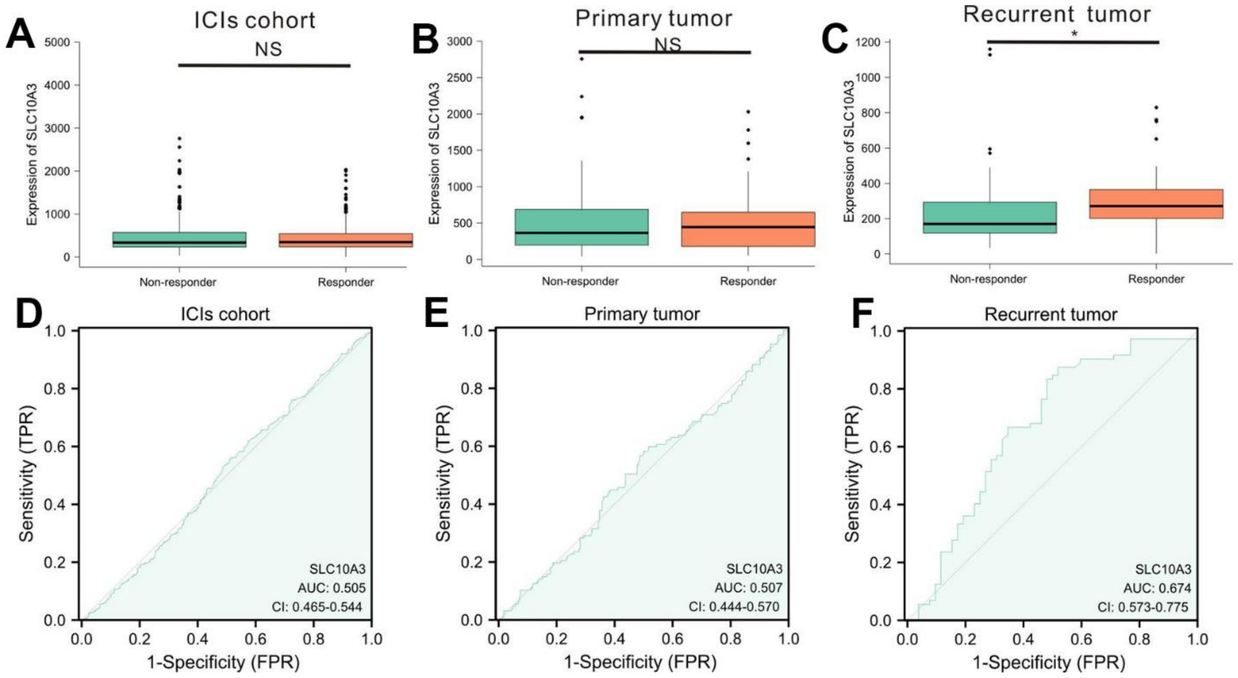

Supplementary Figure 5. Predictive ability of *SLC10A3* to the response of immunotherapy in pan-cancer ICIs cohort. (**A**) There is no significant difference of *SLC10A3* between non-responders and responders in ICIs cohort. (**B**) There is no significant difference of *SLC10A3* between non-responders and responders with primary tumor. (**C**) There is significant difference of *SLC10A3* between non-responders and responders with recurrent tumor. The predictive ability of *SLC10A3* for immunotherapy response is 0.505 for all the cancer population (**D**) 0.507 for the primary tumor individuals (**E**) and 0.674 for the recurrent tumor individuals (**F**).

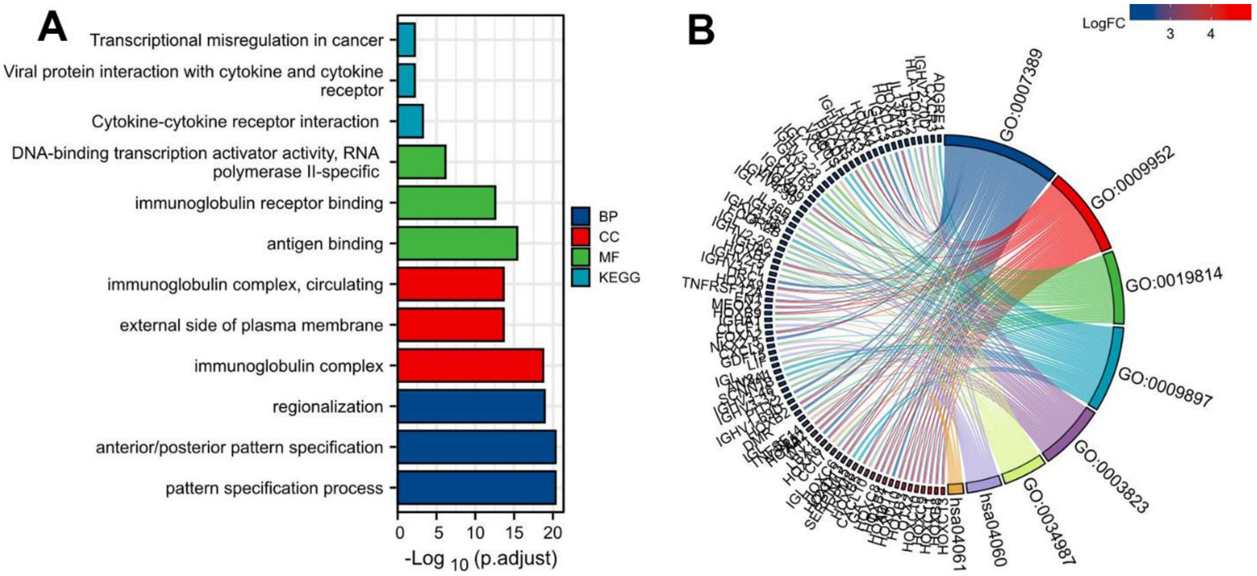

Supplementary Figure 6. Enrichment analysis of *SLC10A3* co-expressed genes in LGG. (**A**) The typical pathways of GO and KEGG analysis of *SLC10A3* in LGG. (**B**) The detailed genes of the most significant GO and KEGG pathways.

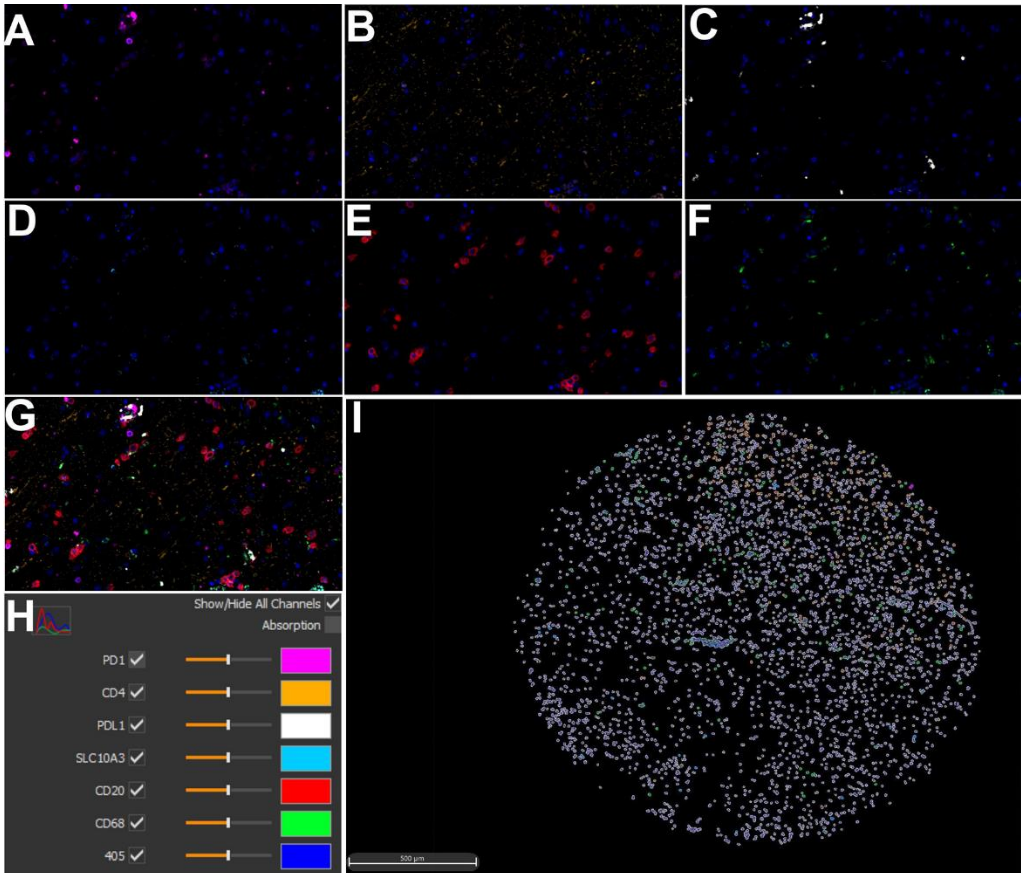

Supplementary Figure 7. Multiplex immunohistochemistry profiling of *SLC10A3* and immune markers in normal brain tissues. (**A**) PD1(pink), (**B**) CD4(yellow), (**C**) PD-L1(white), (**D**) *SLC10A3* (blue). (**E**) CD20(red), (**F**) CD68(green). (**G**) The merged image of seven markers. (**H**) Each marker stands for one special color. (**I**) Cell phenotype image constructed by the seven markers in the multiplex staining.

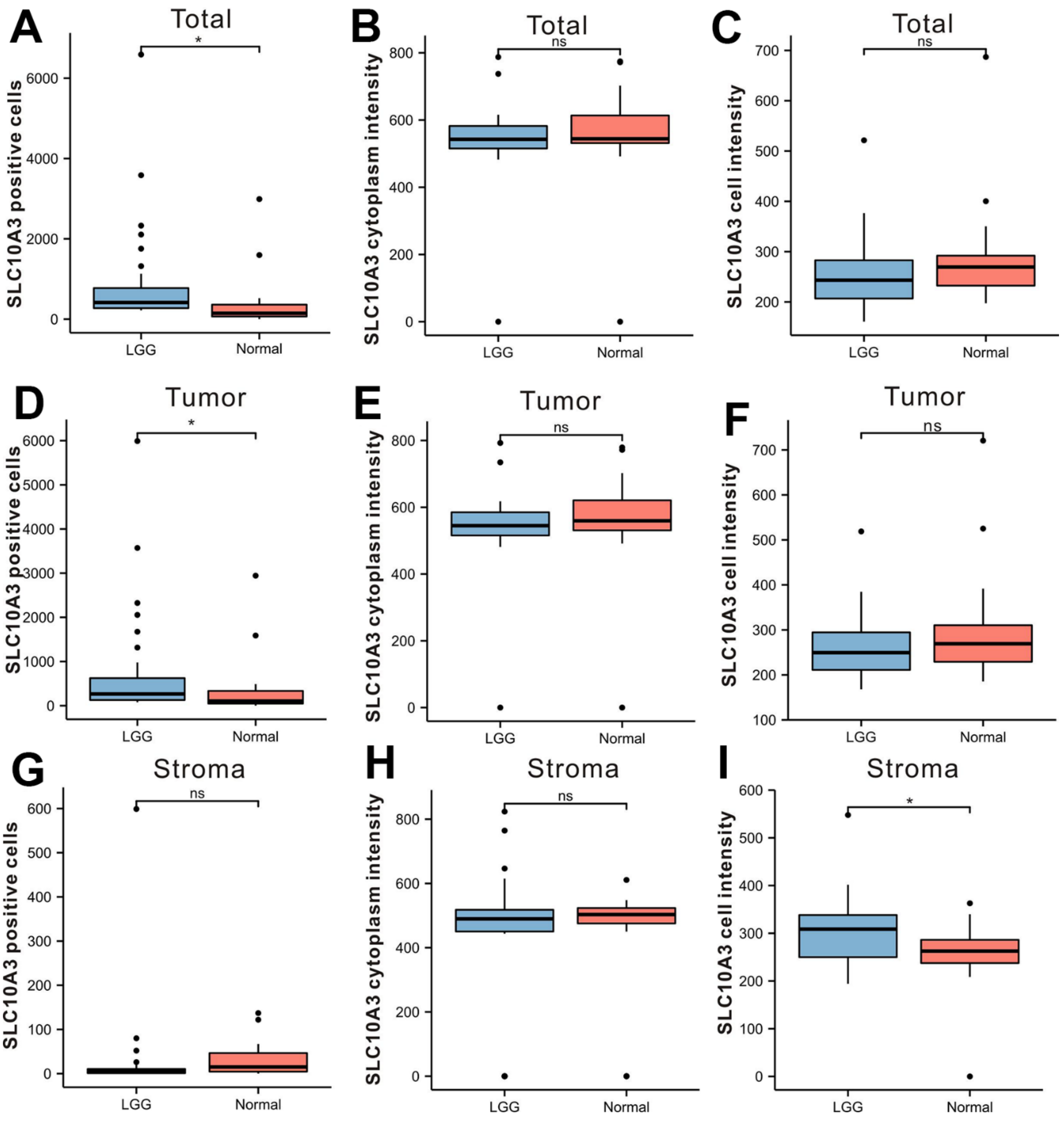

Supplementary Figure 8. Comparison of *SLC10A3* expression between LGG and normal tissues in total area, tumor area and stromal area. (**A**) total *SLC10A3* positive cells, (**B**) total *SLC10A3* cytoplasm intensity, (**C**) total *SLC10A3* cell intensity. (**D**) tumor *SLC10A3* positive cells, (**E**) tumor *SLC10A3* cytoplasm intensity, (**F**) tumor *SLC10A3* cell intensity. (**G**) stromal *SLC10A3* positive cells, (**H**) stromal *SLC10A3* cytoplasm intensity, (**I**) stromal *SLC10A3* cell intensity.
