# Supplementary material for: The prognostic significance and immune correlation of SLC10A3 in low-grade gliomas revealed by bioinformatic analysis and multiple immunohistochemistry
Source: Aging (Albany NY). 2023 May 10;15(9):3771–90. doi: 10.18632/aging.204712 (PMC10449292; doi:10.18632/aging.204712)
Supplement: Supplementary Tables [file aging-15-204712-s002.pdf]

Supplementary Tables

Supplementary Table 1. Multiple covariates Cox regression of overall survival of LGG individuals.

Immune cells	HR	95%CI	P value
B_cell	2.696	0.009-777.626	0.731
CD8_Tcell	53.174	0.06-47226.07	0.251
CD4_Tcell	0.028	0.000-61.505	0.361
Macrophage	309.482	5.617-16940.402	0.005
Neutrophil	0.003	0.000-4.014	0.112
Dendritic	4.76	0.111-203.213	0.415
SLC10A3	2.302	1.242-4.266	0.008

Supplementary Table 2. The detailed information of the primary and second antibodies in mIHC assay.

Antibody	*SLC10A3*	CD68	CD4	CD20	PDL1	PD1
No.	ab224438	ZM0060	ZM0418	ab78237	13684S	ZM0381
Species	rabbit	mouse	mouse	rabbit	rabbit	Mouse
Concentration	1:1000	1:500	1:100	1:100	1:100	1:100
Incubation condition	37° C 1hr	37° C 1hr	4° C ON	37° C 1hr	37° C 1hr	37° C 1Hr
Second antibody	BD(1:3)	BD(1:3)	BD(1:3)	BD(1:3)	BD(1:3)	PV-8000
Dye (1:100)	XTSA 480	XTSA 520	XTSA 570	XTSA 620	XTSA 690	XTSA 780
